# Cardiac function analysis with cardiorespiratory-synchronized CMR

**DOI:** 10.1186/1532-429X-18-S1-W24

**Published:** 2016-01-27

**Authors:** Lennart Tautz, Li Feng, Ricardo Otazo, Anja Hennemuth, Leon Axel

**Affiliations:** 1 grid.428590.20000000404968246Fraunhofer MEVIS, Bremen, Germany; 2 grid.137628.90000000121698901NYU School of Medicine, New York City, NY USA

## Background

Conventional cine MRI provides data on the variation of cardiac dimensions across the cardiac cycle; cardiac function analysis primarily focuses on the difference between end-diastolic (ED) and end-systolic (ES) dimensions of the left and right ventricles (LV and RV). With cardiorespiratory-synchronized (CRS) CMR, there is an additional effective dimension of information available, related to the effect of the respiratory cycle phase on cardiac dimensions. However, there are currently no established ways to analyze this potentially useful additional physiological data. We have developed a set of tools for the functional analysis of CRS CMR, particularly for the study of the respiratory effects on LV-RV interaction, and derived some initial normative values for the results.

## Methods

We have developed a set of interactive CMR function analysis programs. Images from CRS CMR are organized in a two-dimensional matrix, sorted by cardiac and respiratory cycle phases. The user can interactively position an analysis line across the ventricles in a representative image; this line can then be automatically tracked across the other cardiac and respiratory phases. The intensity profile along the line is then used to automatically track the corresponding positions of the edges of the LV and RV free walls and the interventricular septum (IVS). A variety of absolute and normalized variables can be derived from these varying positions, including ED and ES dimensions, and displayed as functional images over the cardiac and respiratory cycle dimensions. CRS CMR was performed with a sparsity-based method (XD-GRASP), using continuous acquisition of radial k-space samples with golden-angle increments and retrospective cardiac and respiratory phase sorting in reconstruction. An initial set of CRS CMR data from 9 normal subjects (age 28.33 ± 5.85) was analyzed, as well as from 3 patients (age 40 ± 9.66, one with HCM).

## Results

On visual inspection of the images, it is apparent that there is a clear shift in the relative position of the IVS over the respiratory cycle, to the left in inspiration and to the right in expiration, reflecting the LV-RV interaction; this is much more prominent near ED than ES. For the normal subjects, in midlevel short-axis views, the respiratory-related absolute shift in IVS position was 1.07-3.23 mm at ED and 0.69-2.14 mm at ES; corresponding values normalized to ED dimension were 2.65-7.08 pp and 1.99-5.18 pp. The ED-ES difference for the normalized shift ranges was -1.9-4.35 pp (median 1.35, first quartile 0.68). For the HCM patient, the difference between the shift ranges was 0.79 pp. Linear regression when plotting NCD against NEDD (reflecting the Frank-Starling relationship and giving an estimate of contractility) was 0.68 ± 0.11 in the normal subjects.

## Conclusions

Novel physiologic data on LV-RV interaction can be derived from CRS CMR; this seems to show consistent ranges in normal subjects, and may provide useful information on disease-related changes in cardiac function.

**Figure 1:**
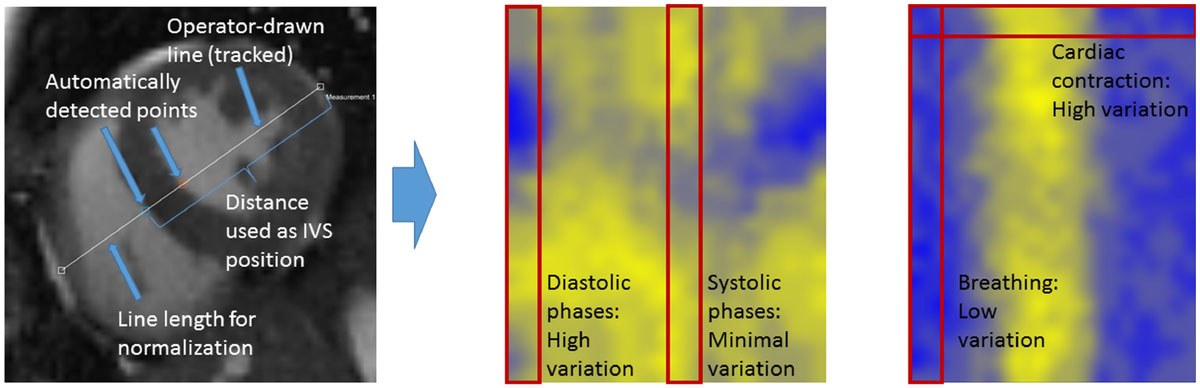
**Left: Position detection concept for the quantitative septum motion assessment**. Middle: Color-coded normalized IVS position for all cardiac and motion states (top-to-bottom: respiratory phase, left-to-right: cardiac phase starting with ED; blue-to-yellow increasing values). Right: Color-coded septum thickness

**Figure 2:**
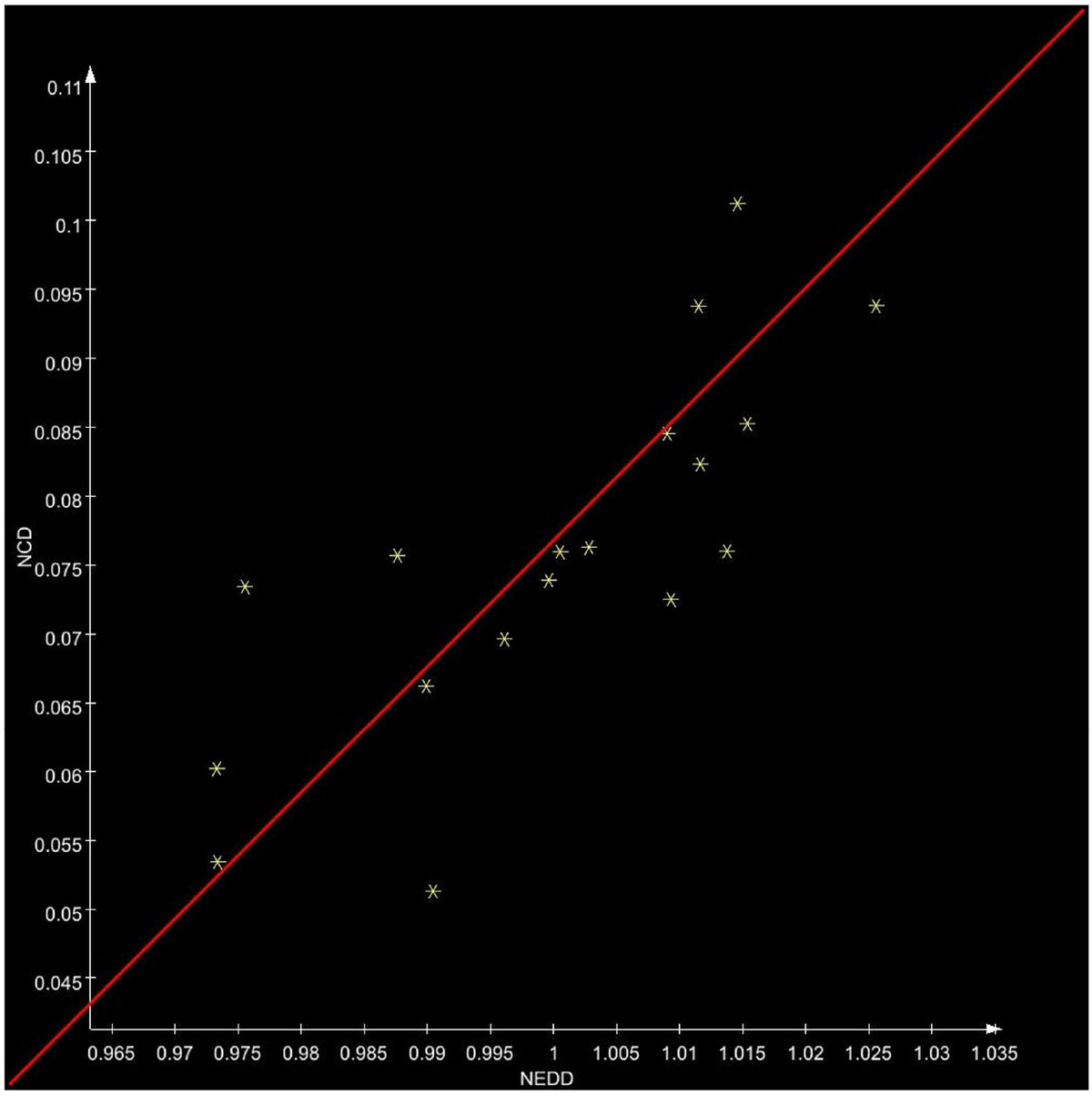
**Representative plot of NCD (normalized change in ventricle dimension) against NEDD (normalized end-diastolic dimension) for all respiratory phases, with linear regression**.

